# Comparison of effectiveness and complications in trabeculotomy with phacoemulsification between *ab externo* and *ab interno* using a spatula-shaped microhook

**DOI:** 10.1038/s41598-021-96701-0

**Published:** 2021-08-26

**Authors:** Satoru Kanda, Takashi Fujishiro, Takashi Omoto, Ryosuke Fujino, Takahiro Arai, Yohei Nomoto, Makoto Aihara

**Affiliations:** 1 Saitama Red Cross Hospital, 1-5 shintoshin, Tyuo-ku, Saitama-shi, Saitama Japan; 2 Department of Ophthalmology, University of Tokyo Graduate School of Medicine, 7-3-1 Hongo, Bunkyo-ku, Tokyo, Japan; 3 Asahi General Hospital, 1326 i, Asahi-shi, Chiba Japan

**Keywords:** Glaucoma, Corneal diseases

## Abstract

To compare the short-term surgical effectiveness and safety profile of trabeculotomy *ab externo* and *ab interno* with microhook in terms of the recovery of visual acuity. A retrospective chart review was performed on patients who underwent trabeculotomy combined with phacoemulsification and lens implantation at Asahi General Hospital, with 6 months of follow-up. The patients treated by trabeculotomy were classified into two groups depending on the surgical procedures: *ab interno* with Tanito microhook (TMH) and *ab externo* with rigid probe trabeculotome (LOT). The demographics, preoperative and postoperative intraocular pressure (IOP), number of medications (Med), best-corrected visual acuity (BCVA), surgical-induced astigmatism (SIA), and postoperative complications were analyzed at pre-operation, and 1 week and 1–6 months post-operation. Fifty-two eyes of 38 Japanese patients underwent TMH and 42 eyes of 32 patients underwent LOT*.* The decreases in IOP and Med from the baseline were significant at all time points in both groups (p < 0.001), but there were no significant differences between the two groups. BCVA improved significantly in TMH and LOT after the operation (p < 0.001). BCVA and SIA significantly improved, mostly at 1 week in TMH, compared with LOT (p = 0.02 and 0.003). Hyphema and IOP spike exceeding 30 mmHg (spike) occurred in 11% and 6% of participants in TMH, and 33% and 26% of participants in LOT, respectively. Hyphema and IOP spike occurred more frequently in the LOT than in the TMH group (p = 0.01 and 0.005). *Ab interno* trabeculotomy showed similar IOP-lowering effects as *ab externo*, but had less postoperative complications.

## Introduction

Glaucoma is a major cause of blindness and vision impairment worldwide^1^, and lowering of intraocular pressure (IOP) with medications or by surgery is essential to treat glaucoma patients. Trabeculotomy is a glaucoma surgery and was first reported by Burian and Smith in 1960. In 1970, Harms subsequently modified the technique, employing a scleral flap to easily identify the Schlemm’s canal (SC), which further popularized the surgery. There are many reports of effective IOP control via trabeculotomy in open-angle glaucoma, including childhood glaucoma, steroid-induced glaucoma, and exfoliation glaucoma^2–5^.

This surgery aims to cleave the trabecular meshwork and inner wall of SC, where the main sight of resistance for aqueous outflow is thought to be^2,6,7^. Conventional trabeculotomy was achieved via the *ab externo* approach with conjunctival incision, which was thought to be a demerit due to a relatively long operation time or conjunctival scarring resulting in obstruction. With the recent development of new devices or implants, minimally invasive glaucoma surgery (MIGS) is emerging as a standard concept^8^. MIGS in outflow surgery reduces the outflow resistance by an approach from the inside of the anterior chamber involving a small incision of the cornea. Thus, the devices or implants for MIGS require a minimally invasive nature and high biocompatibility. Their safety and fast recovery have been evaluated in outflow channel surgeries^9^.

The common MIGS procedures involving SC include the removal of trabecular tissue (e.g.,Trabectome: NeoMedix Corporation,CA,USA, Kahook: New World Medical, CA,USA, Tanito *ab interno* microhook: Inami & Co., Ltd, Tokyo, Japan) or the implantation of a small device into the trabecular meshwork and SC (e.g., iStent, iStent inject, and Hydrus MicroShunt)^10–13^. There have been several studies on the clinical results of these trabecular hooks^14–17^. Tanito microhook is a re-usable hook created by sharpening the tip of a regular Sinskey hook. There are 3 types of this hook; straight, angle-right and angle-left, which allows the surgeons to approach all quadrants of the trabecular meshwork^14^.

Conventional trabeculotomy *ab externo* is still useful in patients with poor corneal transparency. Thus, in the future, both procedures should be use depending on the condition of the eye. There has been only one study on the direct comparison of clinical results of *ab interno* and *ab externo* trabeculotomy^18^. Mori et al*.* concluded that there was no significant difference in IOP and postoperative complications between both methods. However, the recovering transition of VA following hyphema or astigmatism has not been fully investigated. Considering these points, our aims were to compare the surgical effectiveness and safety profile from the early postoperative phase to 6 months, of trabeculotomy, using two methods on Japanese open angle glaucoma patients, combined with cataract surgery.

## Materials and methods

### Subjects

Consecutive series of *ab interno* and *ab externo* trabeculotomy combined with cataract surgery cases between April 2018 and March 2019, performed by four surgeons at the Asahi General Hospital with minimum 6 months of routine follow-up were included. We performed the surgery on patients whose progression we could not control with medications only and who were observed with cataract at the same time. These patients had an indication of trabeculotomy, not trabeculectomy, because of their target pressure of around the middle teen and also their background, such as age. Eyes with past ophthalmic surgeries, such as trabeculotomy, trabeculectomy, goniosynechialysis, argon laser trabeculoplasty, selective laser trabeculoplasty, and vitrectomy, were excluded from the study. When deciding the glaucoma type, we judged a uveitic case with steroid use as steroid glaucoma if we could certainly confirm the IOP reduction after stopping the steroid use. If not, we judged it as uveitic glaucoma. This observational study was approved by the institutional review board of the Asahi General Hospital (Registration number: 2019052116) and conducted in accordance with the principles of the Declaration of Helsinki. All patients read and signed the informed consent for the study in our institute before the surgery. The patients who underwent trabeculotomy were classified into two groups depending on the surgical approach: *ab interno* with Tanito microhook (TMH) and *ab externo* with rigid probe trabeculotome (LOT). The surgical procedure was decided by the period of intervention because TMH had been firstly induced in our hospital from the beginning of 2019. During the first half period, *ab externo* was selected and during the second half, *ab interno* with TMH was selected. All patients had nuclear cataract or cortical cataract of grade II or higher according to Emery-Little classification and simultaneously underwent cataract surgery by temporal scleracorneal incision.

### Surgical technique

In TMH, two corneal ports were made at 1–2 and 10–11 o’clock positions. The inner wall of SC around the inferior 120–150° was cleaved with Tanito microhook through two ports, with assisted gonioscopy. In LOT, a super-blade (Micro feather, Feather Safety Razor co, LTD. Japan) was used to create an inferior temporal scleral flap of 4 × 4 mm. We performed deep sclerotomy and a horizontal cut in the roof of SC. Two rigid probe trabeculotomes were inserted on both sides of the inferior and temporal SC under the flap. The trabeculotomes located in SC were recognized by gonioscopy. The inner wall of SC around the inferior temporal 120° was cleaved with sweeping trabeclotomes into the anterior chamber. Sinusotomy was performed. A sinusotomy is an incision made in both sides of the scleral flap at its attachment to the corneal limbus. Deep sclerotomy and sinusotomy were performed to increase aqueous flow and avoid obstruction due to hemorrhage^19,20^. The flap and conjunctival incision were sutured by 10-0 nylon (Mani, Utsunomiya, Japan). We sutured 2 and 3 stitches in scleral flap and conjunctiva incision respectively. We did not removed sutures in scleral flap. By contrast, we removed sutures in conjunctiva incision at 2 weeks postoperatively. We stopped the antithrombotic therapy at the perioperative phase in both groups. The breakdown of antithrombotic drugs was warfarin potassium, aspirin, rivaroxaban, edoxaban tosilate hydrate, clopidogrel sulfate, and prasugrel hydrochloride. Warfarin potassium, aspirin, clopidogrel sulfate and prasugrel hydrochloride were discontinued 7 days prior to surgery. Rivaroxaban and edoxaban tosilate hydrate were discontinued 24 hours prior to surgery.

### Postoperative examination

A topical antibiotic (levofloxacin) and corticosteroid (betamethasone sodium phosphate) were postoperatively administered 4 times per day, and miotic agent (pilocarpine hydrochloride) 4 times per day. These were reduced step by step and used for a month on average according to postoperative inflammation. IOP-lowering medications were re-started based on the surgeons’ decision.

A retrospective chart review was performed. Observational points were set at 1 week (within ± 2 days) and 1 to 6 months (within ± 1 week) postoperatively. IOP was measured by Goldmann applanation tonometry. The baseline IOP was measured as the final IOP before the surgery. Changes in IOP and number of medications (Med) over time were recorded. Combination drugs were counted as 2 and oral administration of acetazolamide as 1 in the analysis of Med. Postoperative routine use of miotic agents was not counted as IOP-lowering medication. The best-corrected visual acuity (BCVA) measured using a decimal VA chart was converted to the logarithm of the minimum angle of resolution (LogMAR) VA. Changes in VA within 0.2 LogMAR were considered stable.

### Measurement of astigmatism

Preoperative and postoperative astigmatism were recorded at central 3 mm diameter by an autorefractor keratometer (RC5000; Tomey, Nagoya, Japan). These levels and surgical-induced astigmatism (SIA) were calculated from the keratometric values obtained at 1 week, 1 month, 3 months, and 6 months postoperatively using the application of an astigmatism calculator^21^ (SIA Calculator Version 2.1, ^©^2010, Dr. Saurabh Sawhney and Dr. Aashima Aggarwal) based on the vector analysis algorithm^22,23^. Before this analysis, all the keratometric astigmatism values were converted into plus cylinder format. The means of the vector magnitude, vector meridian and arithmetic magnitude of the preoperative and postoperative astigmatism, and SIA values were obtained in each surgical group (TMH and LOT). The distribution of the SIA in each surgical group was visualized on a scatter plot using the Astig PLOT application (Eye Pro 1.0, EB EYE LTD, United Arab Emirates).

### Statistical analysis

Fisher’s exact test, t-test and Dunnett’s test were used appropriately. Reductions of IOP and Med were also analyzed using linear mixed model. Analyses were performed using R (The R version 3.6.1; Foundation for Statistical Computing, Vienna, Austria). Statistical significance was set at p < 0.05.

## Results

Ninety-four eyes of 70 patients were identified. TMH was used in fifty-two eyes of 38 patients (55.3%) and LOT in forty-two eyes of 32 patients (44.7%) for trabeculotomy. The specific patients’ demographics of each group were described in Table 1. Baseline IOP was significantly lower in TMH than in LOT (p = 0.004).

**Table 1:** Preoperative characteristics of patients who underwent trabeculotomy.

	TMH	LOT	P value
No. of eyes (patients)	52 (38)	42 (32)	
Age	75.8 ± 7.42	73.4 ± 7.66	0.08
Female/male	20/18	18/14	
Right/left	29/23	22/20	
**Baseline**			
IOP	16.5 ± 4.6	19.7 ± 5.6	0.004
Medication score	2.6 ± 1.6	3.1 ± 1.7	0.2
Log MAR VA	0.26 ± 0.45	0.34 ± 0.52	0.4
**Indications**			
POAG	33	27	0.2
SOAG	0	4	0.02
PEG	19	11	0.9

Data are presented as mean ± standard division or number.

IOP: intraocular pressure; Log MAR VA: logarithm minimal angle resolution visual acuity; POAG: primary open angle glaucoma; SOAG: secondary open angle glaucoma; PEG: pseudo exfoliation glaucoma.

**Figure 1:**
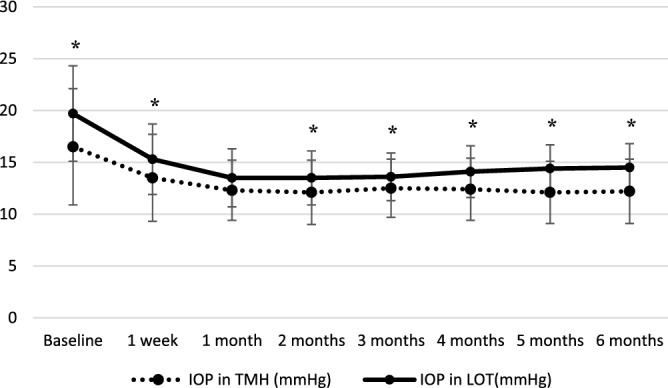
Transition and comparison of IOP. IOP: intraocular pressure. *Significant difference calculated using linear mixed model between TMH and LOT.

**Figure 2:**
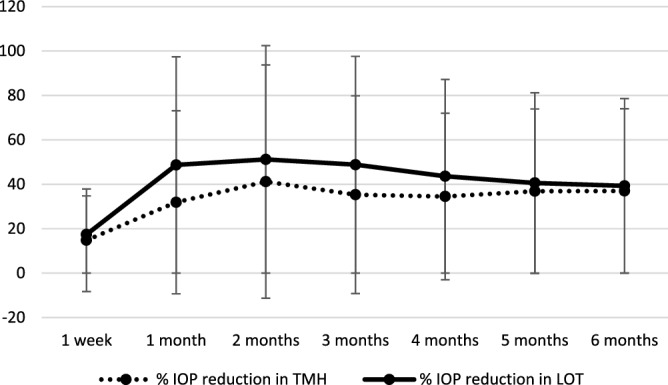
Transition and comparison of rate of IOP reduction. IOP: intraocular pressure. Calculated using linear mixed model between TMH and LOT.

**Figure 3:**
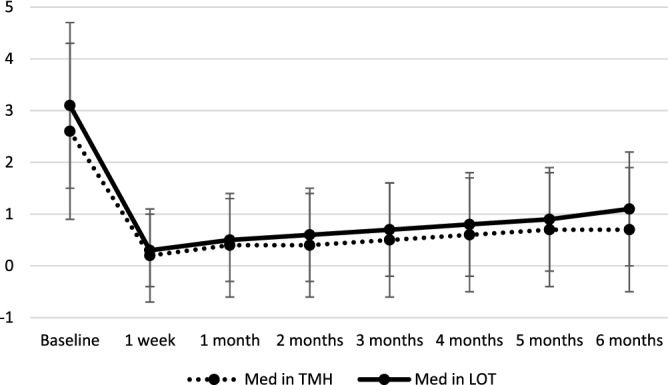
Transition of number of medications. Med: number of medications. Calculated using linear mixed model between TMH and LOT.

Changes in IOP and Med are summarized in Figs. 1, 2 and 3. The decreases in IOP and Med from the baseline were significant at all time point in both groups (p < 0.001). Postoperative IOP was significantly lower in *ab interno* than *ab externo* except at 1 month postoperatively. The reduction rate of IOP from the baseline at each time point was not significantly different between TMH and LOT (p value ranged from 0.1 to 0.8). The reductions of Med were not significantly different at all time points (p value ranged from 0.2 to 0.5).

**Figure 4:**
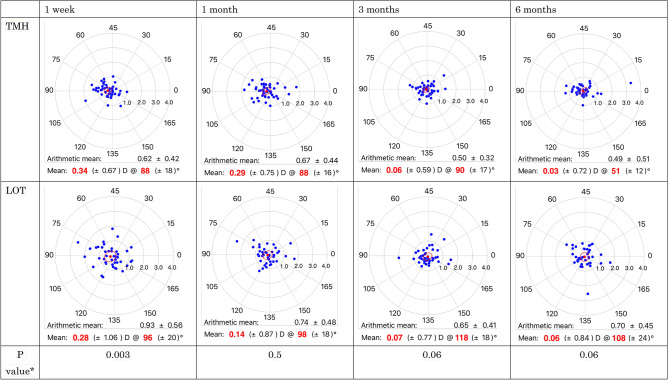
A scatter plot of SIA in TMH and LOT. SIA: surgical induced astigmatism. The red and blue dots in each chart indicate the mean SIA vectors of each group and each time points and the SIA of each eye, respectively. The red circle indicates 95% confidence interval. All the astigmatic values are expressed in a plus-cylinder format. The astigmatism in the direction of 0° indicates against the rule astigmatism, and that in the direction of 90° indicates with the rule astigmatism. Each ring indicates about 1.2 or 1.3D. *Calculated using linear mixed model in surgical induced astigmatism between TMH and LOT.

Changes in LogMAR VA and SIA are summarized in Table 2 and Fig. 4. LogMAR VA improved after operation in both groups. Comparing TMH and LOT, the difference at 1 week was significant (p = 0.02). On the other hand, there was no significant difference at the other timepoints. SIA had a significant difference at 1 week (p = 0.003).

**Table 2:** Transition of visual acuity.

Time point	TMH (n = 52)		LOT (n = 42)		P value **
	Log MAR VA at each time point	P value*	Log MAR VA at each time point	P value*	
Baseline	0.27 ± 0.45		0.35 ± 0.55		
1 week	0.09 ± 0.36	0.05	0.28 ± 0.40	0.7	0.02
1 month	0.09 ± 0.35	0.05	0.09 ± 0.29	0.003	0.9
3 months	0.07 ± 0.33	0.02	0.06 ± 0.25	< 0.001	0.8
6 months	0.08 ± 0.34	0.03	0.06 ± 0.24	< 0.001	0.6

Log MAR VA: logarithm minimal angle resolution.

*Calculated using Dunnett’s test in IOPs between pre- and postoperative values.

**Calculated using linear mixed model in Log MAR VA between TMH and LOT.

We also analyzed the safety profile of trabeculotomies and summarized the results in Table 3. Hyphema with niveau formation (hyphema) and transient IOP spike exceeding 30 mmHg (spike) were two common postoperative complications. Table 3 shows that hyphema and spike were significantly frequent in LOT than in TMH (P = 0.01 and 0.005). Hyphema was seen in all eyes intraoperatively, but no eye needed washout postoperatively. All the patients could be treated with IOP-lowering medications, and none needed additional trabeculectomy during the observational period. The number of cases with both hyphema and IOP spike were significantly more in the LOT group than in the TMH group (P = 0.04).

**Table 3:** Comparison of complication.

	TMH	LOT	P value*
Hyphema with niveau formation	6	14	0.01
Transient IOP spike	3	11	0.005
Spike and hyphema	2	7	0.04
Worsening of VA	1	2	0.4

IOP: intraocular pressure; VA: visual acuity.

*Calculated using linear mixed model in complications between TMH and LOT.

## Discussion

Recently, *ab interno* trabeculotomy is getting more popular using small instruments like MIGS. *Ab externo* trabeculotomy has undergone some changes in the past 50 years. This is the second report to declare the surgical outcome of *ab externo* and *interno* trabeculotomies with microhook, and the first to show the changes in visual acuity with the corneal astigmatism in the early postoperative phase.

In *ab interno*, we used Tanito microhook, which is reported for its effectiveness in previous studies. Tanito et al*.* showed in their report including 68 eyes of various kinds of Japanese glaucoma patients who received *ab interno* trabeculotomy combined with Tanito microhook, that IOP decreased significantly from 16.4 mmHg at baseline to 11.9 mmHg at 6 months with a significantly reduced IOP-lowering medications from 2.4 to 2.1^16^. This report was a retrospective one-arm study of combined procedures. Mori et al*.* investigated the IOP-lowering effect of trabeculotomy using Tanito microhook in 50 eyes of various types of glaucoma patients for 1 year postoperatively. They showed that IOP and Med changed from 28.4 to 17.8 mmHg and from 4.9 to 3.1, respectively^18^. In our study, IOP significantly decreased from 16.5 to 12.2 mmHg (37.0% reduction), and Med decreased from 2.6 to 0.7 (56.7% reduction). Although the baseline IOP and patient background were different among these 3 studies, trabeculotomy *ab interno* using Tanito microhook could significantly reduce IOP and Med. There were previous reports on MIGS with other instruments. One of them suggested that IOP and Med changed from 17.9 to 13.6 mmHg and from 1.9 to 1.0 respectively using the Kahook dual blade^24^. Another previous report revealed that IOP and Med changed from 18.0 to 13.9 mmHg and from 1.7 to 1.1 respectively using Trabectome^25^. Considering these reports, the IOP-lowering effect of Tanito microhook may not be inferior to other MIGS.

In *ab externo*, we used two rigid probe trabeculotomes combined with deep sclerectomy and sinusotomy. Kinoshita et al*.* showed that IOP and Med changed from 24.3 to 16.0 mmHg and from 3.3 to 1.5 after 6 months postoperatively by trabeculotomy combined with cataract surgery, deep sclerectomy and sinusotomy including 59 eyes^26^. Bao et al*.* investigated 25 eyes for 8 years and revealed that IOP and Med changed from 22.2 to 14.8 mmHg and from 4.4 to 3.3 by trabeculotomy combined with deep sclerectomy with the punch out of scleral flap, but without cataract surgery and sinusotomy^27^. Mori et al*.* showed that IOP and Med changed from 32.5 to 18.3 mmHg and 4.3 to 1.7 in 50 glaucoma patients in 1 year after trabeculotomy combined with deep sclerectomy. Cataract surgery was done in 46% of patients in this group^18^. In our study, IOP significantly decreased from 19.7 to 14.5 mmHg (39.3% reduction) and Med changed from 3.1 to 1.1 (52.1% reduction) at 6 months. The postoperative reduction rate of IOP in our study is relatively low compared to past reports, however, considering the low baseline, these results are reasonable, and the Med reduction is comparable to past reports.

In comparison about IOP between the two groups, baseline was significant difference. To compare the effectiveness of the surgery, we employed the IOP reduction rate. When comparing the IOP reduction rates in the TMH and LOT groups, there were no significant differences at all time points. Thus, TMH and LOT had a comparable success rate in terms of postoperative IOP. In a previous study, there were no differences in IOP and Med reduction between *ab interno* and *ab externo* procedures^18^.

In this study, we found that VA in TMH was significantly better than in LOT 1 week after the surgery. Even though there was hyphema, the astigmatism parameters were measured without any problems. We speculate SIA, hyphema and hypotony may be important factors to avoid VA disturbance in the postoperative early phase. Although cases with hyphema at 1 week postoperatively were excluded from the analysis, there was the significant difference between the two groups (p = 0.042). Two patients experienced hypotony (less than 10 mmHg) at 1 week postoperatively. The BCVAs of the two patients were comparable to the preoperative BCVA. Therefore, we thought that the early postoperative VA was affected by SIA although hyphema may be involved. We thought that SIA causes not only regular astigmatism, but also irregular astigmatism as well. Large SIA was thought to be resulted in large irregular astigmatism and contributed to the loss of BCVA. Tanito et al*.* investigated the effect of glaucoma operations on astigmatism and showed that there were no significant differences between *ab interno* and *ab externo* 3 months after the surgery^28^. However, they did not mention about the early postoperative phase.

In the present study, the SIA at 1 week after LOT was greater than that after TMH. However, there was no difference at 1 month and later. The results were consistent with the previous report of Tanito et al. Since the differences in the two surgeries were mainly a making scleral flap with deep sclerotomy and flap sutures, SIA may be induced by the corneal distortion by the scleral flap formation followed by the incision of the supporting limbal tissue. This distortion will reduce due to the postoperative scarring. Furthermore, suturing the flap influences the tension of the sclera and cornea. The tension makes the cornea warp at the early postoperative phase and becomes loose with the lapse of time. The effect for SIA by both factors is inevitable in LOT but is a transient complication ignorable within a month. We analysed the trends for each surgeon. However, there was no trends. The reason was thought to be slightly different angles of scleral flaps even on the same inferior temporal side depending on the surgeon, different flap thicknesses, different suture strengths and directions, and different stiffness of the patient's own cornea and sclera.

Hyphema occurred during the surgery and disappeared gradually in 1–2 weeks postoperatively in almost all the cases. The low frequency of hyphema in TMH than LOT may be the reason for quick recovery of VA in TMH. It is said that hyphema came from the episcleral vein because IOP during and after the surgery fell down suddenly and remained around the episcleral venous pressure. The difference in hyphema occurrence between the two groups may be induced by some factors. The most likely reason is the aqueous flow from the anterior chamber to the outside of the eye in LOT, although a scleral flap and tight flap sutures were performed. In contrast, we cleave the trabecular meshwork and inner wall of SC in TMH. As a result, the outer wall of SC remained intact and there was no direct aqueous route to the exterior of the eye. There is a limitation, hyphema forms niveau and fluttering in the anterior chamber as single red blood cells. We and previous studies count niveau formation as hyphema and do not mention the fluttering type because of difficulties in quantifying. However, the fluttering type hyphema also seems to influence VA. From now on, we have to search a method to quantify fluttering red blood cells.

Hyphema and spike happened as postoperative adverse effects, which were commonly reported in both types of trabeculotomy. In the present study, hyphema in TMH and LOT occured in 11% and 33% of patients respectively. The difference in hyphema occurrence was significant (p = 0.01). The occurrence of hyphema in *ab externo* was 20% (50 eyes), 31% (59 eyes), 93% (44 eyes) and 68% (25 eyes) in the studies by Mori et al*.*, Bao et al., Chihara et al*.* and Kinoshita et al*.,* respectively^18,26,29,30^. Occurrences of hyphema in *ab interno* were 16% (50 eyes) and 41% (68 eyes) in the studies by Mori et al*.* and Tanito et al*.*^16,23^.

One reason for the variation of hyphema occurrence is the definition of hyphema. Our definition was the formation of niveau. Another reason was the cutting angle of trabecular meshwork. Tanito et al*.* originally cut two quadrants (approximately 240°) during the *ab interno* method^16^. However, Mori et al*.* performed two quadrants in the beginning and changed to one quadrant because they experienced considerable hyphema^31^. Our surgery was performed at 120–150° in the TMH and 120° in the LOT. Moreover, we paid attention to maintain the irrigation pressure when we cut the trabecular meshwork to avoid considerable backflow of the episcleral vein. This procedure may have avoided the excessive backflow of blood after the surgery.

Spike occurrence in TMH and LOT were 6% and 26%, respectively, in our study. The difference in spike occurrence was significant between the TMH and LOT groups (p = 0.005). Spike occurrence in the *ab externo* method was 24% (50 eyes), 8% (59 eyes) and 7% (44 eyes) in the studies by Mori et al*.*, Kinoshita et al*.* and Chihara et al*.,* respectively^18,29,30^. Spike occurrence in the *ab interno* method was 36% (50 eyes) and 4% (68 eyes) in the studies by Mori et al*.* and Tanito et al*.,* respectively^16,18^. Our results were comparable with those of the previous studies. The occurrence of spike may be influenced by some factors. One of them is the definition of spike. The definition of Tannito et al*.* was IOP elevation over 30 mmHg^16^. Chiahara et al. defined spike as a sharp IOP elevation over 10 mmHg in 3 days^29^. Our definition was IOP elevation over 30 mmHg. Additionally, because it is difficult to monitor postoperative IOP continuously, we may not have detected the actual IOP spike. The occurrence of spike with hyphema was significant difference in the current study (p = 0.04). The relationship between IOP elevations similar to spikes and red blood cells similar to hyphema had been discussed^32–34^. These reports suggested the possibility that IOP elevation occurred because degenerated red blood cells blocked the flow at the trabecular meshwork. Xu et al*.* showed that the mean IOP elevation at 48 hours after hemorrhage was 46.5 mmHg^34^. Our cases may also have a spike and an IOP elevation with a mechanism similar to that in previous reports. The amount of hyphema may be related to the difference in surgical procedure. In the case of TMH, the anterior chamber temporarily becomes high IOP during anterior chamber washout after cleaving trabecular meshwork, which can aspirate reflux hemorrhage from the episcleral vein and push away hemorrhage in the collector channel. By contrast, the reflex hemorrhage may not have been sufficiently washed out in cases of LOT because the pressure tends to escape due to aqueous humor flowing out of the scleral flap. Therefore, it may be possible that TMH had less spikes due to less reflux hyphema. In our study, there were no eyes needing additional procedure for postoperative complications during the follow-up period.

People who underwent glaucoma surgery tend to be elderly and take some types of the antithrombotic therapy. There are some reports that antithrombotic therapy does not have to be discontinued around the perioperative period. In one of them, the occurrence of hyphema was significantly different but IOP 1 year after the surgery did not differ significantly with and without stopping the antithrombotic therapy. Furthermore, hyphema in the eyes of patients continuing the antithrombotic therapy remained longer than in that of those suspending the therapy, and the former required irrigation of the anterior chamber more. Reduction in IOP and Med 1 year after the surgery was comparable but there are superiorities in safety and complications in the early postoperative phase after stopping the antithrombotic therapy^30^. We mainly stopped the antithrombotic therapy to lower bleeding risk in both groups. However, the increase of elderly patients who must continue the antithrombotic therapy is inevitable. In addition, bleeding risk by conjunctival and scleral incision is inherent in the *ab externo* method. In some patients with low vision, rapid recovery of VA is required to maintain the activeness of daily life. Considering these factors, TMH had better VAs than LOT at the early operative phase because of the low frequency of hyphema and spike in TMH. Thus, we believe that TMH is a quite useful and tolerable method against LOT.

This study has some limitations. This was a non-randomized retrospective case series with a short follow up period. Thus, further studies are needed to evaluate the long-term clinical results and safety profiles after trabeculotomy of *ab interno* and *ab externo*, in a multicenter controlled study. However, the current study provides some empirical support for the decision regarding the proper indications of trabeculotomy.

In conclusion, *ab interno* using Tanito microhook shows a similar IOP-lowering effect as *ab externo*, with less postoperative complications.
